# New Insights into YES-Associated Protein Signaling Pathways in Hematological Malignancies: Diagnostic and Therapeutic Challenges

**DOI:** 10.3390/cancers13081981

**Published:** 2021-04-20

**Authors:** Alessandro Allegra, Giovanni Pioggia, Vanessa Innao, Caterina Musolino, Sebastiano Gangemi

**Affiliations:** 1 Department of Human Pathology in Adulthood and Childhood “Gaetano Barresi”, Division of Hematology, University of Messina, 98125 Messina, Italy; vinnao@unime.it (V.I.); cmusolino@unime.it (C.M.); 2 Institute for Biomedical Research and Innovation (IRIB), National Research Council of Italy (CNR), 98164 Messina, Italy; giovanni.pioggia@cnr.it; 3 School and Operative Unit of Allergy and Clinical Immunology, Department of Clinical and Experimental Medicine, University of Messina, 98125 Messina, Italy; gangemis@unime.it

**Keywords:** YES-associated protein, Hippo signaling, hematological malignancies, multiple myeloma, leukemia, lymphoma, cancer, phosphorylation, apoptosis

## Abstract

**Simple Summary:**

YES-associated protein (YAP) is a co-transcriptional activator that binds to transcriptional factors to increase the rate of transcription of a set of genes, and it can intervene in the onset and progression of different tumors. Most of the data in the literature refer to the effects of the YAP system in solid neoplasms. In this review, we analyze the possibility that YAP can also intervene in hematological neoplasms such as lymphomas, multiple myeloma, and acute and chronic leukemias, modifying the phenomena of cell proliferation and cell death. The possibilities of pharmacological intervention related to the YAP system in an attempt to use its modulation therapeutically are also discussed.

**Abstract:**

The Hippo/YES-associated protein (YAP) signaling pathway is a cell survival and proliferation-control system with its main activity that of regulating cell growth and organ volume. YAP operates as a transcriptional coactivator in regulating the onset, progression, and treatment response in numerous human tumors. Moreover, there is evidence suggesting the involvement of YAP in the control of the hematopoietic system, in physiological conditions rather than in hematological diseases. Nevertheless, several reports have proposed that the effects of YAP in tumor cells are cell-dependent and cell-type-determined, even if YAP usually interrelates with extracellular signaling to stimulate the onset and progression of tumors. In the present review, we report the most recent findings in the literature on the relationship between the YAP system and hematological neoplasms. Moreover, we evaluate the possible therapeutic use of the modulation of the YAP system in the treatment of malignancies. Given the effects of the YAP system in immunosurveillance, tumorigenesis, and chemoresistance, further studies on interactions between the YAP system and hematological malignancies will offer very relevant information for the targeting of these diseases employing YAP modifiers alone or in combination with chemotherapy drugs.

## 1. Introduction

### General Consideration of Hippo/YES-Associated Protein Signaling Pathway

**Figure 1:**
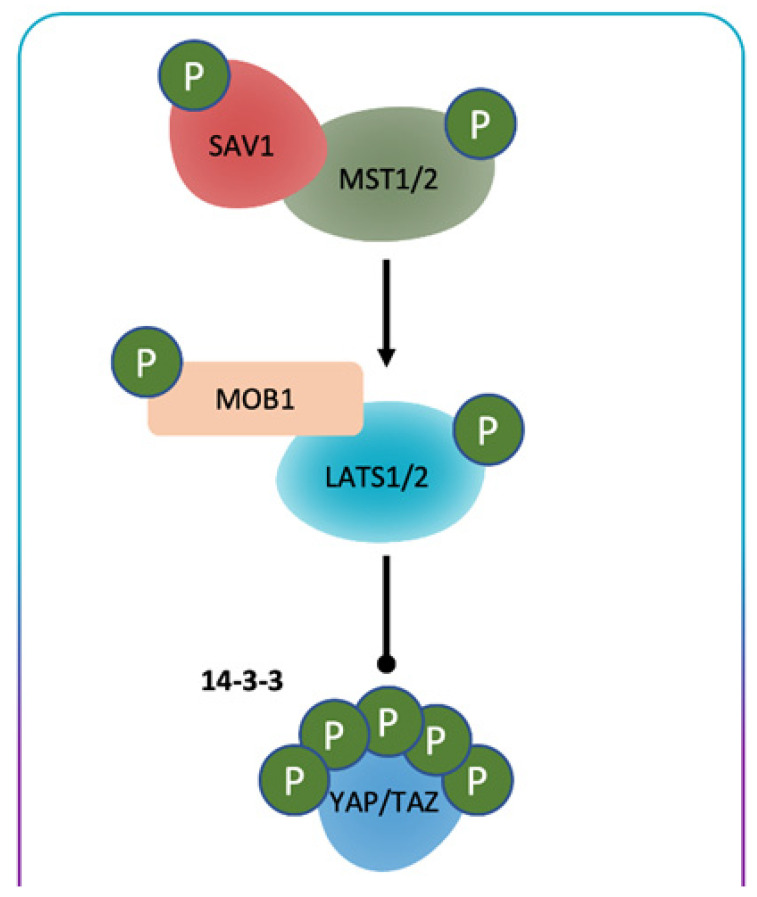
Overview of the Hippo signaling pathway. Hippo signaling is initiated by a variety of upstream stimuli. Activation of Hippo (MST1/2) leads to subsequent phosphorylation of LATS1/2. LATS1/2 negatively regulates the Hippo pathway effector YAP/TAZ. Unphosphorylated YAP/TAZ translocates into the nucleus where it interacts with TEAD transcription factors to upregulate the transcription of a variety of genes. In contrast, phosphorylation of YAP/TAZ leads to its cytoplasmic sequestration by 14-3-3 proteins and degradation.

The Hippo/YES-associated protein (YAP) signaling pathway was initially detected in *Drosophila* sp. It is an evolutionarily preserved proliferation-control system with its main action being the regulation of cell growth and organ volume [1]. YAP is a co-transcriptional coactivator and the more relevant downstream component of the core kinase cascade in this controlling pathway [2] (Figure 1).

The Hippo kinase system in mammals comprises mammalian STE20-like (MST)1/2, large tumor suppressors (LATS)1/2, and their adaptor proteins [3]. After the stimulation of the Hippo pathway by cellular stress due to DNA damage, long-lasting fast, cell–cell contact, or extracellular matrix stiffness, MST1/2 kinases are triggered, which successively phosphorylate the Salvador family WW domain-containing protein 1 (SAV1) scaffold protein and MPS 1 binder kinase activator (MOB)1/2 adaptor protein that supports MST kinase to phosphorylate LATS1 at T1079 and LATS2 at T1041. SAV1 operates to bring LATS and MST together by bonding to both. MOB1 joins to LATS1/2 and provokes the autophosphorylation of these kinases in their triggering loop, which provokes and augments their kinase activity [4]. LATS1/2 can also be phosphorylated and triggered by mitogen-activated protein kinase kinase kinase kinases (MAP4Ks), which operates together with MST to increase the action of LATS1/2 kinases [5,6,7].

Stimulation of MST1/2 manages the phosphorylation of the proliferation-enhancing transcriptional coactivator YAP, provoking the stimulation of activation of successive targets such as cysteine-rich protein 61 (CYR61) and connective tissue growth factor (CTGF) [8].

Activated LATS1/2 phosphorylate WW domain-containing transcription coactivators YAP at five sites and its paralog transcriptional coactivator with PDZ-binding motif (TAZ)/WW domain-containing transcription regulator protein 1 (WWTR1) at four sites [9,10,11,12,13]. Phosphorylation of YAP or TAZ by LATS induces a binding site for 14-3-3 protein that segregates YAP/TAZ in the cytoplasm and blocks YAP/TAZ action (Hippo on) [14]. Moreover, phosphorylation of YAP or TAZ by LATS on other sites can provoke successive phosphorylation by CK1 kinase and consequent destruction by SCF-TrCP E3 ligase [15,16]. However, degradation of upstream elements of the Hippo system and dephosphorylation of the 14-3-3 docking site on YAP or TAZ decrease YAP/TAZ phosphorylation and raise the nuclear passage of YAP/TAZ, thus causing YAP/TAZ stimulation (Hippo off). The stimulated YAP/TAZ can then join to the transcriptional enhancer factor TEF-1 (TEAD family—TEAD1–4), a group of proteins at the C-terminus which have a transactivation domain that binds to YAP/TAZ. In fact, dephosphorylated YAP/TAZ translocates into the nucleus and activates gene transcription through binding to the TEAD (TEA domain) family. Binding of YAP/TAZ to TEAD in the nucleus is required to activate gene transcription. The nuclear TEAD–YAP/TAZ complex is able to recruit the transcription enhancer and mediator, forming a transcriptionally competent complex [17], which can stimulate the transcription of other target genes such as fibroblast growth factor (FGF1), AXL, Bone Morphogenetic Protein 4 (BMP4), and PD-L1 [18,19,20,21,22]. These genes are implicated in carcinogenesis comprising augmented cell growth, decreased cell death, and reduced immuno-surveillance. In addition to the TEAD relationship, YAP/TAZ were also reported to control gene expression by generating a complex with other transcription components such as TBX5, RUNX, and SMADs [23,24,25,26,27].

Investigations into the mechanisms behind YAP/TAZ action have revealed the importance of a direct partnership with chromatin-modifying protein complexes. Specifically, YAP/TAZ have been shown to interact with chromatin-remodeling complexes of the Switch/sucrose nonfermentable family, GAGA factor, mediator complex, and multiple histone methyltransferases to aid in their activation of target gene transcription through modification of DNA packing and organization. Therefore, the effects of YAP/TAZ on target gene activity are context-specific and highly dependent on the interaction with protein complexes capable of remodeling nucleosome positioning in an ATP-dependent manner and imparting posttranslational modifications to histones [28].

Moreover, the Hippo system can react with several signaling pathways such as G-protein-coupled receptors (GPCRs), the WNT pathway, and the PI3K pathway, which can control diverse elements of the Hippo system [29,30,31,32,33].

Therefore, a modified expression of the Hippo system elements can cause anomalous cell proliferation and cancer transformation [34,35,36,37]. However, while several Ser/Thr kinases [38,39,40,41,42,43,44,45,46,47] have been noted to control the Hippo system, the controlling mechanisms of phosphatases in this pathway and their effects on tumorigenesis are less well understood. In any case, the best-known effect of YAP/TAZ action is persistent cell growth via the stimulation of genes involved in DNA replication and repair machinery, transcriptional regulators of the cell cycle, and mitotic kinases [48,49,50,51,52].

## 2. YAP System and Human Cancer

Since its early detection, the Hippo system has been prodigiously proven to have an essential role in tumor diffusion, organ development, immune response, and chemo-resistance. Therefore, clarifying the actions of the Hippo/YAP system in tumor biology and tumor treatment has been one of the most fascinating fields in cancer experimentation [53,54,55].

Usually, when cells grow to an elevated intensity, the Hippo system is stimulated [9], with cytoplasmic retention and subsequent destruction by the ubiquitin proteasome system [56,57]. However, the Hippo system has been reported to be inoperative in cancer cells [58,59,60]. In these cells, YAP cannot be phosphorylated and destroyed efficiently. Unphosphorylated YAP arrives in the nucleus where YAP stimulates transcription factors, modifying the expression of genes implicated in cell growth and programmed cell death [61,62,63]. In fact, as a fundamental effector of the Hippo/YAP signaling system, YAP operates in regulating the onset, progression, and treatment response in numerous human tumors [64]. Nevertheless, several reports have proposed that the effects of YAP in tumor cells are cell-dependent and cell-type-determined, even if YAP usually interrelates with extracellular signaling to stimulate the onset and progression of several sorts of tumors [65,66].

It has been suggested that YAP operates as an oncoprotein [67,68,69,70,71] by networking with TEAD [72], generating a protein complex which is essential for the transcription of genes such as survivin and c-Myc [73,74]. Moreover, the action of YAP/TAZ induces the appearance of chemo-resistant cancer cells and determines a defense against programmed cell death by increasing the production of antiapoptotic genes of the BCL-2 and IAP family, AXL receptor tyrosine kinase, connective tissue growth factor (CTGF), and cysteine-rich angiogenic inducer 61 (CYR61) [10,74,75,76,77]. A different manner via which YAP/TAZ supports cell growth is by modifying cell metabolism. YAP stimulates glucose metabolism by increasing glucose-transporter 3 (GLUT3) [78], guaranteeing the occurrence of an unremitting amount of glutamine for purine and pyrimidine generation by increasing the expression of glutamine synthetase enzyme (GLUL) [79]. YAP also seems able to augment transcription of mTOR system controllers such as L-type amino-acid transporter (LAT1), and TORC1 activator Ras homolog enriched in the brain (RHEB). RHEB is indispensable for the growth of transitory amplifying cells during tissue regeneration, while LAT1 augments the uptake of amino acids [80,81].

Nevertheless, in spite of the reported effects of YAP in the stimulation of pro-growth and antiapoptotic genes, YAP was also noted to operate as a tumor suppressor in specific conditions, whereas it may also demonstrate a proapoptotic effect under particular situations such as DNA injury. It was reported that YAP could augment p73-dependent and ABL1-caused programmed cell death during the DNA-damage procedure [82,83]. YAP can join to the cancer suppressor p73 via the YAP WW domain and the p73 PPPY motif. It was demonstrated that, like p73, YAP is also a substratum of c-Abl (Abelson murine leukemia viral oncogene) and is phosphorylated at Y357 after DNA injury. YAP phosphorylated by c-Abl is stored in greater concentrations, joins to p73, and stimulates proapoptotic targets [84].

In fact, although several reports on the nonreceptor tyrosine kinase c-Abl are dedicated to its action as an oncogene as part of the fusion protein BCRAbl, it is also recognized to have an effect in the DNA injury response [85,86]. As soon as it is activated, c-Abl can stimulate programmed cell death or cause cell-cycle arrest via the phosphorylation of p73, a p53 paralog. Moreover, c-Abl also phosphorylates and blocks the E3 ligase mouse double minute 2 homolog (MDM2), causing stabilization of p53, the principal substratum of MDM2 [87,88]. Recently, c-Abl was reported to operate as a negative controller of epithelial-to-mesenchymal transition and tumorigenic phenotype caused by transforming growth factor-β (TGF-β) in mammary tissue [89]. However, Keshet et al. reported that DNA injury via c-Abl activation decreased YAP–TEAD-caused transcription [90]. c-Abl caused TEAD1 phosphorylation; however, the YAP–TEAD complex remained unchanged. In this context, the post-translational changes in TEAD might have a role. TEAD phosphorylation and palmitoylation have been shown to control its function. TEAD is phosphorylated by protein kinase A and protein kinase C, which have been demonstrated to inhibit TEAD. Recently, TEAD palmitoylation emerged as an essential post-translational regulation mechanism. Protein palmitoylation is critical for protein trafficking and membrane localization. The molecular mechanisms of YAP/TAZ and TEAD regulation through TEAD palmitoylation have been suggested. TEAD palmitoylation did not alter its protein stability. However, it was demanded for the YAP/TAZ interaction. Palmitoylation-deficient TEAD mutants were not able to bind YAP/TAZ. Thus, TEAD transcriptional activity was altered, which implies that TEAD palmitoylation has a relevant effect in controlling its binding to the transcriptional coactivators [91].

TEAD coactivation was altered by phosphomimetic YAP Y357E mutation. Under DNA damage, c-Abl is activated and phosphorylates YAP at the Y357 residue. To evaluate whether the mutations at Y357 affect YAP localization, Keshet et al. expressed different YAP constructs fused to GFP in MCF10 A cells, measuring YAP nuclear localization using FACS. Wildtype YAP and the Y357E and Y357F mutants were all localized mainly in the cytoplasm to the same level. The YAP S127A mutant is resistant to phosphorylation by LATS and accumulates in the nucleus. These findings established that this mutant and the double mutants S127A/Y357E and S127A/Y357F were all localized to the nucleus, suggesting that the Y357 mutation affects neither nuclear localization nor Lats-mediated nuclear exclusion [91]. Moreover, YAP Y357E also altered the effect of YAP in cell transformation and anchorage-independent proliferation in MCF10 A cells. These findings suggest that YAP pY357 lost the TEAD transcription stimulation effect, prove that YAP pY357 blocks YAP oncogenic action, and demonstrate a role of YAP Y357 phosphorylation in cell-fate determination [91]. Thus, in reaction to DNA injury, YAP operates as a tumor suppressor, in contrast to the recognized oncogenic effect of YAP under the other situations reported above.

**Figure 2:**
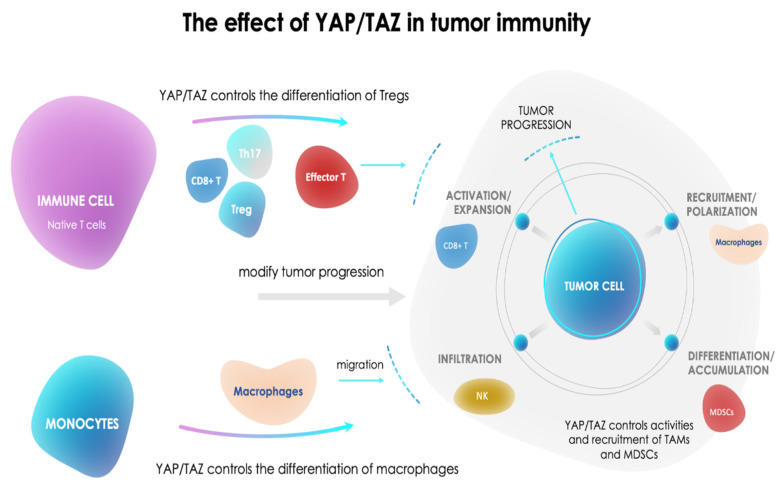
The role of YAP/TAZ in tumor immunity. YAP/TAZ expression in immune cells controls the differentiation and activities of Tregs and macrophages, which change the effector T-cell activity and tumor progression. YAP/TAZ expression in turn controls the activities and recruitment of tumor-associated macrophages (TAMs) and myeloid-derived suppressor cells (MDSCs), contributing to a modification of tumor progression.

However, other mechanisms could intervene (Figure 2) with, in particular, effects exerted on the immune surveillance systems. Pan et al. examined the principal immunological effects of YAP/TAZ in cancer, which eventually guide cancer progression and outcomes [92]. YAP/TAZ levels in immune cells, comprising B, cells, T cells, and macrophages, are able to control the proliferation and activity of immune cells, which are essential for cancer immuno-surveillance. The relationships between the YAP/TAZ system and the immune system appear to be bidirectional. YAP contributes to the suppression of B-cell differentiation by stimulating TEAD2. Activated TEAD2 represses the transcriptional amounts of *cd19* by binding to the 3′-UTR consensus motif of *cd19*, which controls BCR signaling and the differentiation of peripheral B cells. Moreover, YAP/TAZ regulates numerous genes in macrophages, which are closely correlated to cell differentiation, comprising PPARγ, myoblast determination protein, and lymphocyte function-associated antigen 1. Conversely, YAP/TAZ expression in tumor cells indirectly affects the recruitment and activity of tumor-infiltrating immune cells or immune checkpoints through specific signaling pathways to influence tumor progression. YAP stimulation and nuclear localization in tumor cells attract myeloid-derived suppressor cells (MDSCs). MDSCs in turn, intensely inhibit T-cell growth and alter tumor progression [92].

IFN-γ stimulated nuclear translocation and phase separation of YAP after anti-PD-1 treatment in tumor cells. YAP partitioned with the transcription factor TEAD4, the histone acetyltransferase EP300, and Mediator1 and formed transcriptional hubs for amplifying target gene transcriptions, independent of the canonical STAT1–IRF1 transcription program. Disruption of the YAP phase separation decreased tumor proliferation, increased immune response, and sensitized tumor cells to anti-PD-1 therapy [93].

Lastly, a further mechanism for intervention of the YAP/TAZ system in the onset and progression of neoplasms could involve its interference with angiogenesis mechanisms. Solid tumors and hematological malignancies are dependent on vascularization for their growth [94]. In many types of tumors, YAP/TAZ activation is correlated with increased levels of angiogenesis. In addition, endothelial YAP/TAZ stimulation is important for the formation of new blood and lymphatic vessels during development. Several YAP/TAZ downstream targets drive a proinflammatory, hypoxic tumor milieu, causing a self-sustained positive loop of YAP/TAZ activity and tumor angiogenesis. Intervening with these effects is expected to reduce pathological angiogenesis in tumors and decrease tumor proliferation and progression [95].

Lastly, the relationships between the YAP system and noncoding genetic material such as microRNAs (miRNAs), whose role in cancer and hematological neoplastic pathologies is well known [96,97,98,99,100,101], seem to be particularly close.

YAP was reported to control miRNA generation in a cell density-dependent method. It was proposed that deactivation of the Hippo system or the active YAP might be accountable for miRNA modification reported in cancer [102]. In fact, it was demonstrated that nuclear TAZ/YAP is essential to sustain Dicer-caused pre-miRNA management [103,104].

Regarding more specifically the relationship between the YAP system and solid neoplasms, recently, analysis of more than 9000 samples of 33 diverse types of tumor from The Cancer Genome Atlas demonstrated that the Hippo system is one of the signaling systems more commonly modified in tumors. Remarkably, it was reported that there was oncogenic stimulation of YAP1, TAZ, and TEAD2 with amplification in about 17% of cervical cancers, 16% of lung tumors, 15% of esophageal cancers, and 14% of head and neck carcinomas [105]. Moreover, an increase in YAP/TAZ genes was reported in a huge range of subjects affected by ovarian, liver, breast, and oropharyngeal tumors [106]. Lastly, YAP has been recognized as an oncoprotein increased in cholangiocarcinoma, colorectal cancer, prostate, and gastric cancer [24,107,108,109,110,111,112].

## 3. YAP and Hematopoiesis

YAP/TAZ are considered the main controllers of cell proliferation and tissue development [113]. More specifically, YAP/TAZ are stimulated in somatic stem cells to sustain self-renewal and pluripotency and, when ectopically present, can modify the fate of terminally differentiated cells into a stem cell/progenitor-like condition [114,115]. It has been suggested that YAP/TAZ are involved in the control of the hematopoietic system, both in physiological conditions and in hematological diseases.

In cord blood-derived human hematopoietic stem cells (HSCs), transcriptional enhancer factor domain 1 (TEAD1) controls differentiation into early B cells. However, TAZ, but not YAP1, is involved in the lineage selection during naïve T-cell differentiation, where it operates as a coactivator of RORγt supporting TH17 differentiation [116]. This may also explain the effects of the mammalian sterile 20-like kinase (MST) kinases, which are YAP/TAZ inhibitors, in autoimmune diseases [117,118].

To evaluate the effect of YAP and TAZ in the hematopoietic system, Donato et al. employed an animal experimental model. They crossed mice double homozygotes for conditional knockout alleles of both YAP and TAZ (Yapflox/flox/Tazflox/flox mice) with Mx1-CRE mice. Their findings propose that YAP/TAZ are essential for adult hematopoiesis and HSCs self-renewal [119]. This confirms analogous experiments performed in other somatic cells [120]. However, these results do not exclude the effects of YAP/TAZ on lineage-restricted functions [121], although it is undeniable that YAP is a stem-cell controller [122,123] and is present in the stem-cell population [124].

Nevertheless, while in solid tumors, YAP/TAZ are emerging as either powerful oncogenes or targets of oncogenic systems, while their action in hematopoietic malignancies seems to be context-dependent. In multiple myeloma (MM) and leukemias, YAP appears to have a tumor-suppressive effect by controlling the Abl1-dependent DNA damage response, which causes programmed cell death in tumor cells. This explains why a deletion of or reduction in YAP/TAZ is generally reported in MM and leukemias [84]. However, TEADs have been suggested to support the transcriptional stimulation of oncogenic programs during B-cell transformation [125], and several Hippo elements are lost in leukemias and lymphomas [126,127], thus suggesting a pro-oncogenic effect of YAP/TAZ on some hematological neoplasms.

In the next section, we report the most recent findings in the literature on the relationship between the YAP system and hematological neoplasms (Table 1).

## 4. YAP and Hematological Malignancies

### 4.1. YAP and Lymphoproliferative Diseases

Diffuse large B-cell lymphoma (DLBCL) is the most diffused form of lymphoma, accounting for 25–35% of non-Hodgkin lymphoma (NHL) cases [142]. DLBCL is distinguished by elevated heterogeneity in gene expression, clinical progression, and outcome. Alterations in gene expression provoke a modified stimulation of the signaling system and pathways, as well as heterogeneity in treatment response [143,144].

Particularly interesting, both to illustrate the mechanisms of action of the YAP system on the disease and to be able to hypothesize a possible therapeutic intervention, are the studies aimed at evaluating the correlation with other protumor pathways. One study reported the relevant effect of YAP on the genesis of DLBCL and revealed the controlling action of insulin-like growth factor-1 receptor (IGF-1 R) on the Hippo/YAP system, proposing a new, possible therapeutic approach to cure DLBCL. An amplified expression of YAP was remarkably associated with lymphoma progression and a bad outcome. A knockdown of YAP expression reduced cell growth and caused cell-cycle arrest in DLBCL cells [128].

Verteporfin (VP), a benzoporphyrin derivative, employed as a photosensitizer for age-related macular alteration [145], was reported to alter the communication between YAP and TEAD independent of light stimulation [146]. Several findings indicate that VP can block YAP expression in different malignancies [147,148] and has an antitumor action by modulating the expression of YAP and its target genes, such as CYR61 and CTGF. In vitro (human DLBCL cell lines: LY1, LY8, LY3, and Val) and in vivo experimentations (on 5 week old female beige mice with severe combined immunodeficiency) demonstrated that the reduction in YAP presence with a CRISPR/Cas9 genome-editing system considerably contained tumor progression. Moreover, the reduction in IGF-1R expression provoked a sizable diminution in YAP expression. On the contrary, the addition of IGF-1 increased YAP expression [128] and upturned the block of YAP provoked by IGF-1R inhibitors. The growth of DLBCL cell lines was reduced by IGF-1R inhibitors in a concentration-dependent manner. The use of an IGF-1R inhibitor and of chemotherapeutic substances in refractory tumors is being proven in numerous trials [149,150,151,152] and could represent an interesting new therapeutic approach for DLBCL via their action on the YAP system.

Natural killer T-cell lymphoma (NKTCL) is a form of aggressive Epstein–Barr virus NHL whose morbidity is positioned first for T-cell lymphoma. NKTCL originates from mature NK cells and NK-like T cells, and it is characterized by highly aggressive progression, adverse outcome, and brief survival [153].

The preceding results reported that MST1 was markedly present in hematopoietic cells, and that knockdown of MST1 could induce the onset of lymphoma by determining chromosomal instability [126]. In an experiment, the expression of MST1 was reduced in NKTCL tissues and cell lines (NKTCL samples and cell lines SNK-6 and YTS), while the presence of YAP was remarkably augmented, and the phosphorylation of YAP was blocked [129]. Augmentation of MST1, knockdown of YAP, or VP administration could reduce cell growth, as well as stimulate cell-cycle arrest and programmed cell death in NKTCL cells, while knockdown of MST1 and augmentation of YAP stimulated cell growth. Moreover, Bcl-2/Bax ratio and other effectors of Hippo signaling system such as CTGF, survivin, c-myc, cyclin D 1, and TEAD were remarkably reduced when MST1 was increased and YAP was knocked down after VP administration. Moreover, an animal experimental model (6 week old male BALB/c nude mice) established that stimulation of the Hippo system through increasing MST1 or reducing YAP reduced the tumorigenesis of NKTCL [129]. In this study, increased expression of MST1 and knockdown of YAP could reduce the Bcl-2/Bax ratio and other survival-correlated compounds in NKTCL cells, thus modulating the growth and cell death of NKTCL cells. YAP can network with transcription elements, such as AP-1, SMADs, TBX5, and p73, or with transcriptional controllers, such as the ICD of ERBB4, among which TEADs are the principal transcriptional elements controlling the tumorigenic action of YAP [130,154]. Liu-Chittenden et al. reported that the porphyrin components, such as VP, hematoporphyrin, and protoporphyrin IX, can be used as YAP inhibitors to block the contact between YAP and TEADs and could reduce cell proliferation [155].

Lastly, the YAP mRNA expression in subjects affected by mantle cell lymphoma (MCL), chronic lymphocytic leukemia (CLL), and splenic marginal zone cell lymphoma (SMZCL) was much greater than that in healthy controls. The expression amount of LATS1 and LATS2 mRNA in MCL was associated with molecular cytogenetic alterations, progression-free survival (PFS), and overall survival (OS). The LATS1 expression amount was significantly minor in MCL patients who presented detection P53 compared to subjects without detection, and the LATS1 expression amount was minor in the dead subjects than the survival subjects. The PFS and OS were greater in MCL subjects with a greater amount of LATS1 than in other MCL subjects. Lastly, the Hippo system is also functionally altered in B-NHL, particularly in MCL. The altered expression of LATS1 and LATS2 in MCL subjects is correlated with aggressive disease and might be a relevant prognostic factor [156].

### 4.2. YAP and Multiple Myeloma

In spite of relevant innovations in treatment, multiple myeloma (MM) remains an incurable tumor [157,158,159,160,161].

YAP is habitually hyper-stimulated in MM [92]. Although the main cell death system in MM is due to the BCL-2 family correlated mitochondrial pathway, DNA instability was demonstrated to provoke programmed cell death via the ATM–ABL1–YAP1–p73 signaling pathway, which is also p53-autonomous [162,163,164]. MM cells decrease the YAP1 amount by stimulating the generation of the serine threonine kinase STK4. Both in vitro and in vivo MM experimental studies have demonstrated that STK4 block re-establishes the YAP1 amounts and provokes MM programmed cell death. Recently, the employment of a different YAP1 activator in the MM IM-9 cell line stimulated programmed cell death and reduced cell proliferation [131]. Sirtuin 6 was also reported to reduce the Hippo system and support aging of MM cell lines, while sirtuin reduction could cause MM cell death [131].

A different possibility for the HIPPO system to interfere with MM cell proliferation is correlated to the mitophagy process. Mitophagy, the procedure of mitochondrial autophagy, is caused by modifications in mitochondrial DNA or mitochondrial membrane depolarization [165]. Mitophagy defends against the delivery of proapoptotic components, production of noxious reactive oxygen species (ROS), and futile hydrolysis of adenosine triphosphate by damaged or depolarized mitochondria [166].

Several genes are involved in the onset of mitophagy. PTEN-induced putative kinase 1 (PINK1), a mitochondrial serine/threonine kinase, and Parkin/Parkin RBR E3 ubiquitin protein ligase (PARK2), an E3 ubiquitin ligase, operate as principal controllers of mitophagy [167,168]. The amount of *PINK1* and *PARK2* in CD138^+^ plasma cells is decreased in MM subjects and associated with clinical prognosis in MM patients. Furthermore, the stimulation of PINK1-dependent mitophagy with salinomycin or carbonylcyanide-*m*-chlorophenylhydrazone or an augmented expression of PINK1 provokes a reduction in myeloma cell homing and reduced osteolytic bone lesions. A study performed on several types of cells (MM.1 S, MM.1 R, and U266) demonstrated that the effects of PINK1-dependent mitophagy on MM onset are regulated by the stimulation of the MPS 1 binder kinase activator (MOB1) B-mediated Hippo pathway and the successive reduction in YAP/TAZ expression [132].

Interestingly, elements of the Hippo system have been reported to be abnormally present in CD138^+^ cells of relapsed MM patients. Although the YAP1 amount did not appear to be an independent prognostic factor for OS, these experiments demonstrated the possible existence of a new mechanism of MM resistance to therapy which would be worth investigating [169].

The Hippo/YAP system could, however, be important, not only in the onset and progression of myelomatous disease, but also in the determinism of bone complications, principally concerning osteoblast control [136]. In fact, cytoplasmic YAP has been reported to connect to the APC/β-catenin complex and stimulate its proteasomal destruction; thus, it counteracts Wnt signals supporting osteogenesis [133]. However, nuclear YAP may interrelate with nuclear β-catenin, subsequently causing a persistent augmentation of β-catenin concentrations in the nucleus and unremitting preosteogenic action [138].

Moreover, in vitro experiments performed in chondrocyte cell lines have demonstrated that YAP1 negatively modulates chondrocyte differentiation [170]. Microarray findings from distraction osteogenesis experiments in animals have shown that the Wnt and Hippo pathways are strongly involved in this condition [171]. Furthermore, the Hippo elements such as the YAP/TAZ–TEAD complex, MST2, Aiuba, and RASSF2 seem to be correlated to the modulation of osteoclastic maturation and activity [137].

Li et al. reported that TAZ expression is remarkably decreased in the blood samples of MM subjects with respect to healthy controls [172]. Moreover, TAZ has been recognized as a coactivator of the transcription factor Runx2, which provokes the differentiation of mesenchymal stem cells to osteoblasts [173]. In osteoblastic MC3 T3-E1 cells exposed to different dosages of bortezomib, it has been demonstrated that TAZ inhibition in the MM milieu is essentially due to FGF-2 (fibroblast growth factor 2) [174]. Remarkably, bortezomib blocks FGF-2 independently of proteasomal inhibition and re-establishes TAZ concentrations, thereby stimulating the generation of Runx2 which regulates osteoblastogenesis [174]. Moreover, blockage of proteasome activity by bortezomib may avoid the proteasomal degradation of the cytoplasmic YAP/β-catenin complex and support preosteogenic Wnt signals.

A further, different intervention mechanism of the Hippo/YAP system on MM can, however, be hypothesized. As reported above, a controlling system of YAP activity is represented by the miRNA system. Circular RNA (circRNA) is a type of specific noncoding RNA that has a closed-loop configuration [175]. About 80% of circRNAs are cytoplasmic circRNAs that control gene expression through sponging miRNAs [176]. In fact, it has been reported that circRNA operates in multiple modes, but the best recognized activity is that of the miRNA sponge, in which circRNA is capable of decreasing the inhibitory effects of miRNA on its target gene [177]. Several experiments have demonstrated that circRNA contributes to tumor onset and chemo-resistance by sponging miRNA [178,179].

A new circRNA, circ-CDYL, has been identified as a novel element able to stimulate cell growth [180]. Chen et al. reported that circ-CDYL was increased in MM cells and blood samples and evaluated the mechanism of its tumorigenic action [177]. Experiments have demonstrated that circ-CDYL increased the DNA synthesis of MM cells (MM1.S and NCI-H929) and blocked programmed cell death. Cytoplasmic circ-CDYL was placed together with miR-1180, and circ-CDYL sponged miR-1180 to increase YAP expression, thus supporting MM progression. Significantly, they further demonstrated the presence of this circ-CDYL/miR-1180/YAP controlling system in vivo by employing a xenograft tumor experimental model (BALB/c nude mice). In this report, YAP was decreased in circ-CDYL knockdown cells, and miR-1180 silencing rescued this action, demonstrating that this axis of circ-CDYL/miR-1180/YAP is present in MM cells [181]. These results confirm that circ-CDYL is a tumorigenic element in MM, which stimulates MM cell proliferation via modulation of the miR-1180/YAP pathway, and they lead to speculation of the possibility that circ-CDYL may be a possible target for a therapeutic approach in MM patients.

Other types of noncoding material, such as long noncoding RNA (lnCRNA), could also have a regulatory effect on the YAP system in MM. It is well known that, in MM cells, lncRNA MALAT1 targeting miRNA-509–5p could control the expression of Forkhead box protein P1 (FOXP1) to stimulate the progression of MM [134], and in vivo analyses performed on male athymic BALB/c mice, 5 weeks old, demonstrated that increased lncRNA MALAT1 in blood samples of MM subjects is correlated with an adverse outcome [182,183,184].

One study assessed the effect of lncRNA MALAT1 on the growth of MM cells and whether or not lncRNA MALAT1 has a controlling action via the Hippo/YAP signaling system [135]. LncRNA MALAT1 was augmented in MM cells, and lncRNA MALAT1 interference was able to block cell growth and stimulate programmed cell death in vitro (myeloma cell lines U266, MM.1 S, RPMI8226) and in vivo (male nude mice). Moreover, lncRNA MALAT1 interference could inhibit cell adhesion through the Hippo/YAP signaling pathway [135].

### 4.3. YAP and Acute Myeloid and Lymphoblastic Leukemias

YAP expression was evaluated in numerous leukemia cell lines, and increased YAP expression was reported in Jurkat cells, an immortalized line of human T lymphocyte cells employed to analyze acute T-cell leukemia [139]. To further investigate its action on Jurkat cells, the lentivirus transduced short hairpin RNA (shRNA) method was utilized to block YAP. As expected, the YAP-specific shRNA radically reduced YAP expression at the mRNA and protein levels. Decreased leukemia cell growth and augmented programmed cell death were reported in YAP knockdown Jurkat cells. It was also established that YAP knockdown provoked an abnormal expression of a group of genes essential for cell growth or death, comprising protein kinase B, B-cell lymphoma 2 (BCL2), and BCL2 like protein 1 [139]. A reduction in YAP expression seems to have an essential effect on Jurkat cell growth and death, and it could be used as a possible therapeutic target.

Chen et al. evaluated a different type of leukemia and determined the effect of YAP on the onset of acute promyelocytic leukemia (APL) [140]. They assessed the consequences of YAP knockdown or inhibition employing shRNA or VP, respectively. Knockdown or inhibition of YAP provoked a cell-cycle block at the G0/G1 phase, reduced cell growth, augmented concentrations of Bax and cleaved PARP, and reduced concentrations of Bcl-2, survivin, PARP, and cyclinD1. Moreover, Hoechst 33,342 staining demonstrated augmented cell nuclear fragmentation [140]. These data demonstrate that a blockage of YAP reduces growth and causes programmed cell death in HL-60 cells. Therefore, a new therapeutic protocol implicating block of YAP could be attempted for APL.

In a different study, an APL cell line involving NB4 cells was exposed to VP for 24 h [141]. The authors found that VP reduced the growth of NB4 cells and increased programmed cell death in a concentration- and time-dependent manner. These effects were due to the ability of VP to cause a reduction in the protein expression of YAP, p-YAP, c-Myc, cyclinD1, survivin, p-ERK, and p-AKT. Moreover, VP augmented the protein generation of cleaved caspase3, Bax, cleaved PARP, and p-p38 MAPK [141].

An alteration of the YAP system was also reported in the in vivo studies. In acute myeloid leukemia (AML), augmented expression of the LATS2 gene was identified in 32 de novo AML subjects, indicating that LATS2 may be correlated with leukemogenesis [185]. Another group discovered that AML subjects with t (8;12) translocation presented the MST2-ETV6 fusion gene, which is a potential oncogene [186], while in acute lymphoblastic leukemia (ALL) subjects presenting t(12;21) chromosome translocation, an essential element of the Hippo system called KIBBRA was reported to be greatly methylated and could be the more relevant underlying leukemogenesis factor in this type of leukemia [187]. The LATS2 gene is also a methylation modification target hotspot in ALL subjects; its minor expression due to methylation predicts subjects’ adverse outcome [188].

Lastly, a modification of the YAP system could also be of interest with respect to the resistance to chemotherapeutic treatment of leukemia. It was reported that AML cells displayed less vulnerability to adriamycin (ADR) in the presence of hypoxia, while blocking hypoxia-inducible factor 1α (HIF-1α) via CdCl2 can render AML cells again susceptible to ADR, even under hypoxia [189]. However, hypoxia or stimulation of HIF-1α can remarkably augment YAP expression in AML cells, and resistant cells display a great concentration of YAP, which may not only increase HIF-1α stability, but also stimulate HIF-1α activity on the target gene pyruvate kinase M2. HIF-1α or YAP may constitute a therapeutic objective for overwhelming resistance toward adriamycin-based treatment in AML [189].

### 4.4. YAP and Chronic Myeloid Leukemia

Chronic myeloid leukemia (CML) is a clonal disease distinguished by BCR/ABL, a constitutively activated tyrosine kinase created from the reciprocal translocation between chromosomes 9 and 22.

Although it has been established that YAP-caused programmed cell death is due to the abnormal presence of ABL1 in the nucleus in MM cells [84], in CML cells, where ABL1 was usually translocated, the activity of YAP is less clear. However, it is possible that it exerts its effects through an action on specific target genes [190,191,192,193].

Moriyama et al. evaluated the phosphorylation condition of YAP and demonstrated that it was constitutively phosphorylated at tyrosine 357 in two CML cell lines (TCC-S and K562) [194]. Administration of imatinib (IM) or RK-20449 reduced cell proliferation and reduced tyrosine phosphorylation of YAP in both CML lines. Expression of cyclin D1 or survivin was reduced in TCC-S. These data propose that BCR–ABL causes tyrosine phosphorylation of YAP probably via Src family kinases, which induces expression of survivin and cyclin D able to cause leukemogenesis in CML cells.

In a different experiment, Chorzalska et al. evaluated the effects of miRNA-181a inhibition in a cellular model of CML cells exposed to IM [195]. CML cells displayed a decrease of Hippo signaling and an increased expression of YAP. They were able to demonstrate the alteration of nine out of 52 Hippo core interactors and 146 out of 715 Hippo pathway interactors in K562-STI-R cells.

As we have great concentrations of YAP and TAZ, one would presume YAP/TAZ target genes to be remarkably increased [196,197,198]. In the data, it was only reported that augmented transcript concentrations of CTGF may be correlated with the process via which YAP and TAZ control expression. Both pass to the nucleus and bind to different transcription factors, thus permitting the transcription of target genes. These transcription factors may be lacking or inactive in IM-resistant cells. Moreover, blocking of miRNA-181a employing a microRNA sponge inhibitor caused a reduced stimulation of YAP and heightened sensitivity to IM. These results may suggest that long-lasting exposure to IM provokes an alteration of stem-cell renewal/regulatory Hippo/YAP signaling, with attainment of expression of stem-cell markers, and that interference with YAP action may aid to re-establish chemo-sensitivity to IM.

The existence of an important correlation between YAP and CML has also been demonstrated by other in vivo studies. One report assessed the expression amounts of genes encoding YAP, *LATS1, LATS2*, and *TAZ* in CML subjects at diverse phases of the disease that were resistant or sensitive to IM treatment and in healthy controls. The expression amounts of the target genes were connected to the CML Sokal’s prognostic score. The most remarkable results were the *LATS2* increased expression in CML subjects, the augmented expression of *TAZ* in CML subjects at advanced phases, and the increased *TAZ* expression in CML IM-resistant patients [199].

These findings confirm that *LATS2* is augmented in CML subjects with respect to normal subjects. LATS1/2 operate in the control of DNA injury causing programmed cell death by phosphorylating YAP and TAZ and then avoiding their passage to the nucleus. As reported above, *LATS2* reduces DNA injury-caused programmed cell death in high-cell-density media by blocking the Abl tyrosine kinase, which is a relevant cell death inducer under DNA damage [200,201]. In CML subjects, *LATS2* may have a diverse action in Bcr–Abl-positive leukemic cells participating in their apoptosis resistance phenotype. It was also reported the *YAP* presented an increased expression in CML subjects in advanced phases and in patients who were resistant to IM treatment. CML subjects, who responded to IM therapy and displayed complete cytogenetic remission after 12 months, presented minor concentrations of the *YAP* gene expression. These findings propose that increased *YAP* also contributes to CML progression and resistance to TKI phenotype in Bcr–Abl leukemic cells [202,203].

The development of drugs for the Hippo/YAP signaling pathway can improve CML therapy by augmenting the vulnerability of leukemic cells to programmed cell death and driving a better outcome. As shown later, evidence has suggested that VP can inhibit cell proliferation and increase the efficacy of IM in chronic myeloid leukemia [203].

## 5. Possible Therapeutic Use of the Modulation of the YAP System

Owing to the rising evidence showing the tumor suppressor effect of the Hippo signaling system and alteration of YAP/TAZ in tumor onset in different cell types, it is essential to analyze the substances which can affect the Hippo pathway and control YAP/TAZ proteins to cure solid and hematological malignancies. In this section, we discuss the different types of drugs which can target Hippo signaling. Although most of these compounds have been used in experiments aimed at evaluating their effects in solid neoplasms, it is likely that many of them could be used in hematological neoplasms.

The compounds that can block YAP/TAZ signaling can be separated into three different groups. The first group comprises drugs operating on controllers that can modify YAP/TAZ functions and interfere with the activity of other signaling pathways which can modulate Hippo signaling. Instead, compounds of group II work directly on YAP/TAZ or TEADs and annul their interfaces. Lastly, drugs in group III are usually directed at one of the oncogenic transcriptional gene targets of YAP/TAZ [204] (Table 2).

Recently, the discovery of protein–protein interaction disruptors (PPIDs) that interrupt the YAP/TAZ–TEAD collaboration has gained significant impetus. Numerous experiments have confirmed that YAP/TAZ are no longer oncogenic when their interaction with the TEAD transcription factors is blocked. Peptidomimetic modalities, such as cystine-dense peptides and YAP cyclic and linear peptides, manipulate surface pockets on TEADs and operate as PPIDs. However, the TEAD surface might create a problem for producing an efficacious small-molecule PPID, but TEADs also have a central pocket that is different from the surface pockets, which small molecules leverage exclusively to alter the YAP/TAZ–TEAD communication (allosteric PPIDs). In spite of certain progress, more selective PPIDs that also exhibit advantageous pharmacokinetic characteristics and demonstrate a supportable toxicity profile are essential to assess the clinical possibility of employing these PPIDs for tumor treatment [208].

Among the most studied inhibitors of YAP, the best known is verteporfin (VP), mentioned above several times. Porphyrin family components such as VP, hematoporphyrin (HP), and protoporphyrin IX (PPIX) have been reported as inhibitors of YAP [146,147,148]. In vitro and in vivo studies have demonstrated that inhibition of YAP by VP synergistically reduces tumor cell proliferation when in combination with drugs such as 5-FU [209].

Src homology region 2-containing protein tyrosine phosphatase 2 (SHP2) inhibitors have gained great consideration by both blocking tumor cell growth and stimulating T-cell immune responses toward tumor cells. In one study, authors reported the discovery of an allosteric SHP2 inhibitor 1-(4-(6-bromonaphthalen-2-yl)thiazol-2-yl)-4- methylpiperidin-4-amine (compound 23) which changes SHP2 in a closed structure by binding to the interface of the *n*-terminal SH2, C-terminal SH2, and phosphatase domains. Compound 23 inhibits MAPK signaling and blocks YAP transcriptional effects, demonstrating antitumor efficacy in in vivo studies [210]. The findings suggest that allosteric inhibition of SHP2 could be a practicable line for tumor treatment.

Cottini et al. suggested a model whereby lymphoma, MM, and leukemia cells have persistent DNA injury with nuclear localization of ABL1 [211]. However, programmed cell death does not succeed due to genetic deactivation or decreased concentrations of YAP1. Reduction of the kinase STK4 restores YAP1 and stimulates programmed cell death, giving justification for the identification of new STK4 inhibitors for clinical use in the treatment of hematologic malignancies. Moreover, since this system does not appear to be dependent on p53, it might also be possible to attain a clinical advantage in the subjects displaying p53 inactivation, which is often correlated with a negative outcome.

A different possibility might be the opportunity of affecting the YAP system employing phytochemicals or naturally occurring compounds. Curcumin, a polyphenol from the roots of *Curcuma longa* [212,213], was reported to decrease the generation of YAP/TAZ in tumor cells, further inhibiting the tumor onset and progression [214,215]. A different compound is Decursin, extracted from the roots of *Angelica gigas*, which has been demonstrated to augment destruction of YAP by increasing the level of phosphorylated LATS1 and β-TrCP in tumor cells [216]. Apigenin, a flavonoid extracted from fruits and vegetables, was also recognized as able to decrease YAP/TAZ action and the expression of their target genes in cancer cells [217]. Lastly, Cucurbitacin B, a triterpene substance derived from Cruciferae and Cucurbitaceae was reported to increase LATS1 generation and reduce the expression of YAP, CYR61, and c-myc in tumor cells [218].

A completely different approach could be to intervene in the mevalonate (MVA) pathway. MVA has a main action in isoprenoid biosynthesis. The substrate, 3-hydroxy-3-methyl-glutaryl-CoA (HMG-CoA), is transformed to MVA by 3-hydroxy-3-methyl-glutaryl-CoA reductase (HMGCR). MVA can be also transformed to farnesyl pyrophosphate (FPP), geranylgeranyl pyrophosphate (GGPP), or other molecules. FPP and GGPP are two essential elements regulating post-translational prenylation of the small GTPases [219,220].

Lately, the MVA pathway has become a possible therapeutic target in tumor therapy [221,222]. For instance, atorvastatin, an HMG-CoA reductase inhibitor, is able to block K562 and HL60 cell growth, cause G2/M cell-cycle arrest in K562 cells, and stimulate programmed cell death in both cell lines. Atorvastatin is able to alter the YAP system, while inhibition of YAP nuclear localization and stimulation by Atorvastatin was upturned by adding mevalonate, GGPP, or FPP. Furthermore, the actions on cell-cycle arrest- and programmed cell death-related proteins by Atorvastatin were reduced by addition of mevalonate, indicating that the antileukemia action of Atorvastatin might be via the mevalonate–YAP axis in K562 and HL60 cells [205]. These findings imply that Atorvastatin might be employed for leukemia treatment; however, confirmation of clinical effectiveness is mandatory. Moreover, it is remarkable that the dosages of Atorvastatin employed in this experiment greatly outdo those employed for cardiovascular disease treatment. However, seeing its pronounced antileukemia action and small collateral effects, further experiments involving drug combinations with other antileukemia drugs with decreased dosage merit being performed.

Lastly, the irreversible ERBB1/2/4 inhibitor neratinib provokes mislocalization of K-RAS; this action is increased by HDAC inhibitors. The combination of neratinib and HDAC inhibitors was found to destroy lymphoma T cells. Neratinib degrades MST4 via autophagy that reduces membrane stiffness and is essential for the inactivation of YAP/TAZ signaling [223,224,225].

In the future, other therapeutic approaches seem conceivable. For instance, combining the Hippo pathway controllers with targeted treatments against RAF and MEK or immune checkpoint inhibitors in order to avoid the occurrence of resistance to the latter appears a promising option [226,227], and clinical trials including drugs modulating the YAP system are currently ongoing [206,207].

## 6. Conclusions and Future Perspectives

A well-adjusted control of protein phosphorylation by kinases and phosphatases is essential in practically all biological activities [228]. However, although alteration of protein phosphorylation provoked by kinases or phosphatases participates in several diseases comprising tumors, the molecular mechanisms of phosphatases are rather less recognized with respect to kinases.

This is particularly true for the Hippo/YAP pathway, whose action in the determinism of hematological neoplasms is made more complex by the fact that it appears to be cell- and context-dependent.

A further problem is that since the vast majority of studies on YAP activity have been performed in adherent cells, where Rho-mediated actin cytoskeleton polymerization is needed to promote YAP nuclear translocation, it should be clarified how suspended cells, such as leukemia lymphomas, could bypass the need for actin polymerization to promote YAP nuclear translocation. In fact, YAP/TAZ is regulated by a complex system involving cell- and tissue-level structural characteristics such as the mechanical features of the cell microenvironment comprising tissue stretching, elasticity of the extracellular matrix, organization of cell–cell junctions, and tissue polarity, as well as cell-autonomous elements [229]. In addition to these elements, YAP/TAZ activity can be controlled by cell metabolism and by extracellular signaling pathways. Moreover, control of YAP/TAZ also comprises shear stresses exerted by blood flow [230]. With respect to regulation of YAP/TAZ in cultured cells, at least three different systems have been suggested, namely, stimulation of LATS1/2, closure of the nuclear pores due to rounding up of the nucleus, and inhibition of nuclear F-actin, which allows the nuclear actin-binding factor ARID1 A to sequester YAP/TAZ away from TEADs [231,232].

It is probably still too early to expect a clinical employment of drugs against YAP/TAZ to treat hematological malignancies, and numerous problems will have to be resolved before this can happen. In fact, it is reasonable that blocking the YAP/TAZ signaling pathway might cause toxicity [233,234,235,236,237].

A different, yet important problem is the detection of neoplastic subjects who respond to treatment with a YAP/TAZ inhibitor. YAP/TAZ expression is small in nontumor cells, while their concentrations are remarkably increased in tumors. However, there is no absolute certainty that an increase in YAP or TAZ in tumor cells may constitute a conclusive criterion for effective employment of a YAP/TAZ inhibitor. YAP and TAZ might not be transcriptionally operating nor be drivers in these tumor cells. Moreover, they could influence genes that negatively control their action [235]. The hematological malignancies need to be precisely evaluated to ascertain if they are caused by YAP/TAZ modifications. Moreover, to potentiate the effects of YAP/TAZ inhibition and overwhelm a possible bypass system or the onset of drug resistance, a combined administration of group I and II drugs could also be employed, and a combined blockage of multiple downstream target genes could be contemplated if they are mutually critical for oncogenic activity of YAP/TAZ-driven transcription.

However, given the effects of YAP system in immuno-surveillance, tumorigenesis, and chemo-resistance, further studies on the interactions between the YAP system and hematological malignancies will offer relevant information for the targeting of these diseases employing YAP modifiers alone or in combination with chemotherapy drugs.

## Figures and Tables

**Table 1:** Principal interactions between YAP system and hematological malignancies.

Hematological Malignancies	Yap Status	Mechanisms	Therapeutic Approach	Study	Ref.
Diffuse large B-cell lymphoma	Amplified Expression	Effects on cell proliferation and cell cycle arrest	Verteporfin (VP), CRISPR/Cas9 genome editing system, IGF-1R inhibitors	In vivo and in vitro	[128]
Natural Killer T–Cell Lymphoma	Amplified expression (phosphorylation blocked)	Effects on Bcl-2/Bax ratio, CTGF, surviving, c-myc, cyclin D 1, AP-1, SMADs, TBX5, p73, ICD of ERBB4	Effects on Bcl-2/Bax ratio, CTGF, surviving, c-myc, cyclin D 1, AP-1, SMADs, TBX5, p73, ICD of ERBB4	In vivo In vitro In vitro	[126] [129] [130]
Multiple Myeloma	Reduced levels in MM cells (hyper-stimulated)	Generation of the serine ethreonine kinase STK4, stimulation of MOB1B, effect on mitophagy Effect on circ-CDYL/miRNA-1180 (reduced apoptosis) LncRNA MALAT/MiRNA-181a-5p (reduced apoptosis). Action on bone disease (APC/β -catenin complex, Wnt signal, generation of Runx2, fibroblast growth factor 2	STK4 block, Sirtuin reduction. miRNA-1180 silencing; LncRNA MALAT1 interference. Bortezomib	In vitro In vitro and in vivo In vitro In vitro and in vivo In vitro	[92,131,132] [133,134,135] [136,137] [138] [137]
Acute T cell leukemia	Increased YAP expression	Augmented leukemia cell growth and reduced programmed cell death (effects on protein kinase B, B-cell lymphoma 2 and BCL2 like protein 1)	Lentivirus transduced short hairpin RNA method	In vitro	[139]
Acute promyelocytic leukemia	Increased expression	Reduced concentrations of Bax and cleaved PARP, increased levels of Bcl-2, survivin, PARP, and cyclinD1. Possible effects on c-Myc, survivin, p-ERK, p-AKT, cleaved caspase3, and p-p38 MAPK	YAP knockdown or inhibition (shRNA or VP)	In vitro	[140,141]
Chronic myeloid leukemia	Constitutively phosphorylated	Effects on Src family kinases, survivin and cyclin D	Imatinib mesylate, RK-20449, block of miRNA-181a, VP	In vitro In vivo and in vitro	[194,195,199] [203]

**Table 2:** YAP/TAZ-inhibiting drugs.

Groups	Targets	Substances	Effects	Ref.
Group I	Upstream proteins, SFKs, AMPY, Phosphatases, EGFR, CPCR, Integrins, Adenylyl cyclase families, gamma-secretase, ErbB signaling, ILK	Kinase inhibitors, MEK/MAK inhibitors (trametinib, CAY10561, FR180204), Gamma secretase inhibitors, Epigenetic modulators (Panobinostat, Dacinostat, vorinostat), Actine modulators (Blebbistatin, ML-7, Cytochalasin D, Latrunculin A), Phosphatases inhibitors (okadaic acid, Calyculin A), SK2 inhibitors (Dasatinib, PP2, SV6656, AZ D0530), PI3 K inhibitors (BX795, Wortmannin/LY294002, Temserolimus, MLN0128), Mevalonate pathway inhibitors (statins, zoledronic acid), Cellular stress modulators (metformin, phenformin), AICAR. Erlotinib, AG-1478, Losartan, Dihydrexidine, Gallein, Clengitide, Agrin, RGD peptides, VEGFR inhibitors (Apatinib, SU4312, Axitinib, Pazopanib), Forskoli, Theophylline, IBMX, Odulilast, Rolipran, Dibenzapine, QLT0267, FAK inhibitors (PF-562271, PF-573228, CT-707)	Activate YAP and TAZ, promote TAZ degradation	[204,205,206,207]
Group II	Disruptors preventing the formation of the YAP/TAZ-TEAD complex	Verteporfin, YAP cyclic peptide (peptide 17), cystine-dense peptide (TB1 G1), Vgll1–4, substances targeting TEADs’ palmitate-binding pocket (fenamate derivatives, vinylsulfonamide derivatives, K-975)	Target either TEAD family of transcription factors or YAP/TAZ	[146,147,148,204,208,209]
Group III	Downstream YAP/TAZ targets: metabolic enzymes (aldehyde dehydrogenase, aspartate transaminase, cyclooxygenase 2), kinases, ligands and proteins (BCL-xL, FOXM1, TG2)	A37, celecoxib, WZ400, CXCL5 neutralizing antibody, SB255002, Jagged-1 neutralizing antibody, deoxybouvardin, CYR61 (093 G9) antibodies, navitoclax, thiostrepton, NC9	Inhibition of proteins that are expressed under YAP/TAZ influence	[204]
